# Secretion, Maturation, and Activity of a Quorum Sensing Peptide (GSP) Inducing Bacteriocin Transcription in Streptococcus gallolyticus

**DOI:** 10.1128/mBio.03189-20

**Published:** 2021-01-05

**Authors:** Anthony Harrington, Alexis Proutière, Ryan W. Mull, Laurence du Merle, Shaynoor Dramsi, Yftah Tal-Gan

**Affiliations:** a Department of Chemistry, University of Nevada, Reno, Reno, Nevada, USA; b Unité de Biologie des Bactéries Pathogènes à Gram-positif, Institut Pasteur, Paris, France; c CNRS UMR2001, Paris, France; d Université de Paris, 75013, Paris, France; University of Illinois at Chicago; University of Minnesota Medical School

**Keywords:** *Streptococcus*, bacteriocins, peptide, quorum sensing

## Abstract

Streptococcus gallolyticus subsp. *gallolyticus* is an emerging opportunistic pathogen responsible for septicemia and endocarditis in the elderly. Invasive infections by S. gallolyticus subsp. *gallolyticus* are strongly linked to the occurrence of colorectal cancer (CRC). It was previously shown that increased secondary bile salts under CRC conditions enhance the bactericidal activity of gallocin, a bacteriocin produced by S. gallolyticus subsp. *gallolyticus*, enabling it to colonize the mouse colon by outcompeting resident enterococci (L. Aymeric, F. Donnadieu, C. Mulet, L. du Merle, et al., Proc Natl Acad Sci U S A 115:E283–E291, 2018, https://doi.org/10.1073/pnas.1715112115). In a separate study, we showed that S. gallolyticus subsp. *gallolyticus* produces and secretes a 21-mer peptide that activates bacteriocin production (A. Proutière, L. du Merle, B. Périchon, H. Varet, et al., mBio 11:e03187-20, 2020, https://doi.org/10.1128/mBio.03187-20). This peptide was named CSP because of its sequence similarity with competence-stimulating peptides found in other streptococci. Here, we demonstrate that CSP is a bona fide quorum sensing peptide involved in activation of gallocin gene transcription. We therefore refer to CSP as GSP (gallocin-stimulating peptide). GSP displays some unique features, since its N-terminal amino acid lies three residues after the double glycine leader sequence. Here, we set out to investigate the processing and export pathway that leads to mature GSP. Heterologous expression in Lactococcus lactis of the genes encoding GSP and the BlpAB transporter is sufficient to produce the 21-mer form of GSP in the supernatant, indicating that S. gallolyticus subsp. *gallolyticus* BlpAB displays an atypical cleavage site. We also conducted the first comprehensive structure-activity relationship (SAR) analysis of S. gallolyticus subsp. *gallolyticus* GSP to identify its key structural features and found that unlike many other similar streptococci signaling peptides (such as CSPs), nearly half of the mature GSP sequence can be removed (residues 1 to 9) without significantly impacting the peptide activity.

## INTRODUCTION

Streptococcus gallolyticus subsp. *gallolyticus*, previously known as Streptococcus bovis biotype I, is an emerging opportunistic human pathogen belonging to the highly diverse Streptococcus bovis/Streptococcus equinus complex (SBSEC) (1–3). S. gallolyticus subsp. *gallolyticus* is responsible for causing infective endocarditis and septicemia and has been consistently associated with colorectal cancer (CRC) (4, 5). Recent experimental data support both a passenger and driver role for S. gallolyticus subsp. *gallolyticus* in CRC development (6–8). Using a murine CRC model, it was shown that CRC-specific conditions strongly promote colonization of the colon by S. gallolyticus subsp. *gallolyticus* (9). Indeed, increased levels of secondary bile salts in tumor-bearing mice enhanced the bactericidal activity of gallocin, a putative class IIb bacteriocin encoded by two genes *gllA1 and gllA2*, produced by S. gallolyticus subsp. *gallolyticus*, thus enabling it to colonize the mouse colon by outcompeting resident enterococci.

It was later reported that S. gallolyticus subsp. *gallolyticus* strain TX20005 secretes a 21-mer peptide that induces the production of unknown bacteriocins that are active against various oral streptococci (10). This peptide was named competence-stimulating peptide (CSP) because of its sequence similarity to other CSPs found in other streptococci. However, under the conditions tested, S. gallolyticus subsp. *gallolyticus* CSP was unable to induce natural competence as measured by plasmid DNA uptake (10).

Originally discovered in Streptococcus pneumoniae, natural competence was shown to be a tightly regulated process involving a hormone-like cell product, termed a pheromone (11). The molecule inducing competence in pneumococci was identified as a linear unmodified 17-residue peptide named CSP (12). CSP, encoded by *comC*, is synthesized as a precursor peptide of 41 residues containing the Gly-Gly consensus processing site found in peptide bacteriocins (13). CSP is secreted and maturated by a specialized ATP-binding transporter, ComAB, that cleaves its N-terminal part just after the Gly-Gly motif (14). Once CSP has reached a threshold concentration in the extracellular medium, it binds to a transmembrane histidine kinase receptor, ComD, which in turn triggers phosphorylation of ComE, a response regulator that activates the transcription of *comX*, the master regulator of competence genes (14, 15). *comX*/*sigX* encodes an alternative sigma factor allowing the coordinated expression of a set of approximately 20 genes encoding the competence machinery. This *comABCDE* quorum sensing (QS) circuitry has been found to regulate competence in 12 streptococcus species belonging to the mitis and anginosus groups (16–18). In 2010, two groups showed that natural competence could be induced in a wide range of streptococci by a second QS circuitry involving a 7-amino-acid peptide called XIP (for *sigX*/*comX*-inducing peptide) (19, 20).

Importantly, competence and bacteriocin production in streptococci are often coupled processes that are regulated differentially by the two QS circuitries described above (21). For example, the *comABCDE* circuitry in Streptococcus mutans regulates the production of bacteriocins called mutacins directly and competence through activation of the *comRS* circuitry (14, 22–25). This observation has led to the proposal that the S. mutans CSP be renamed mutacin-inducing peptide (MIP) (14, 26, 27). Additionally, it was shown that the secreted 21-mer CSP/MIP peptide is inactive and requires further processing by the streptococcal extracellular protease (SepM), which cleaves the three C-terminal residues of 21-mer CSP/MIP to generate the active 18-mer CSP/MIP (28, 29).

S. gallolyticus subsp. *gallolyticus* possesses both the *comABCDE* and *comRS* loci in its genome (30–32). In the accompanying paper (33), it is demonstrated that the S. gallolyticus subsp. *gallolyticus* CSP 24-mer is a bona fide QS-inducing peptide involved in activation of gallocin transcription. However, despite several attempts, we failed to induce competence in S. gallolyticus subsp. *gallolyticus* with CSP (9, 10, 33). We therefore propose referring to S. gallolyticus subsp. *gallolyticus* CSP as GSP (gallocin-stimulating peptide) and renaming its putative transporter associated genes, currently called *comAB* and *comDE*, as *blpAB* and *blpHR* (bacteriocin-like peptide histidine kinase and regulator, respectively). Since GSP is predicted to be a 24-mer peptide while the isolated GSP was found to be a 21-mer peptide starting three residues after the double glycine leader sequence of the precursor peptide, we first tested the activity of GSP 24-mer versus the GSP 21-mer on gallocin transcription using a green fluorescent protein (GFP) reporter construct described in the accompanying paper (33). Next, we set out to explore how GSP was processed and evaluated the potential role of S. gallolyticus subsp. *gallolyticus* extracellular protease, which is homologous to SepM. Finally, we undertook a comprehensive structure-function analysis of the GSP pheromone, which revealed that almost half of the peptide sequence (first nine N-terminal residues) is dispensable and can be removed without significantly affecting GSP’s ability to activate its cognate histidine kinase receptor, providing a minimal structure for the development of GSP-based QS inhibitors that could affect S. gallolyticus subsp. *gallolyticus* fitness during competition with the gut microflora.

## RESULTS

### The GSP active form is a 21-mer peptide.

We first aimed at determining the active form of GSP in S. gallolyticus subsp. *gallolyticus* strain UCN34. GSP is derived from a 45-residue precursor encoded by *gsp* and is predicted to be processed to a 24-mer peptide by cleavage of the amino-terminal leader sequence after a double glycine motif and then exported to the extracellular environment by the BlpAB transporter. However, GSP was previously isolated as a 21-mer peptide from S. gallolyticus subsp. *gallolyticus* TX20005 (10). We first confirmed by mass spectrometry analysis that GSP isolated from culture supernatants of strain UCN34 was strictly identical to the 21-mer peptide previously purified from strain TX20005 (Fig. S1). The predicted 24-mer GSP was shown to activate transcription of the gallocin genes (33). Therefore, we chemically synthesized GSP 21-mer and GSP 24-mer peptides to test their efficiency in activating gallocin gene transcription. To do so, we used a reporter S. gallolyticus subsp. *gallolyticus* strain, UCN34, in which the endogenous *gsp* gene has been deleted and which contains a plasmid expressing *gfp* under the control of the gallocin gene promoter (P*gllA*) (33). No expression of *gfp* was observed in the absence of GSP, whereas both 24-mer and 21-mer GSP peptides were able to activate P*gllA*. The GSP 21-mer was found to be more active than the 24-mer, as can be seen from the 50% effective concentrations (EC_50_s) of both peptides (i.e., the concentration of peptide needed to reach 50% of maximal receptor response). Indeed, the GSP 21-mer peptide EC_50_ (2.96 nM) was around 100 times lower than GSP 24-mer EC_50_ (287 nM), indicating that the 21-mer is significantly more active than the 24-mer (Table 1).

**TABLE 1:** EC_50_s of the S. gallolyticus subsp. *gallolyticus* GSP 21-mer and 24-mer in UCN34 Δ*gsp*^*a*^

Compound	Sequence	EC_50_ (nM)^*b*^	95% CI^*c*^
GSP 21-mer	DFLIVGPFDWLKKNHKPTKHA	2.96	1.70–5.14
GSP 24-mer	KNKDFLIVGPFDWLKKNHKPTKHA	287	145–568

a See Materials and Methods for details of reporter strains and methods. All assays were performed in triplicate.

b EC_50_s were determined by testing peptides over a range of concentrations.

c CI, confidence interval.

10.1128/mBio.03189-20.1 FIG S1 LC-MS of UCN34 supernatant after 16 h incubation. S. gallolyticus subsp. *gallolyticus* GSP expected: H_2_^+2^/2 (1246.6855 Da) and H_3_^+3^/3 (831.4594 Da). Download FIG S1, PDF file, 0.3 MB. Copyright © 2021 Harrington et al. 2021 Harrington et al. This content is distributed under the terms of the Creative Commons Attribution 4.0 International license.

### An ABC transporter is responsible for the secretion of GSP and gallocin peptides.

**FIG 1:**
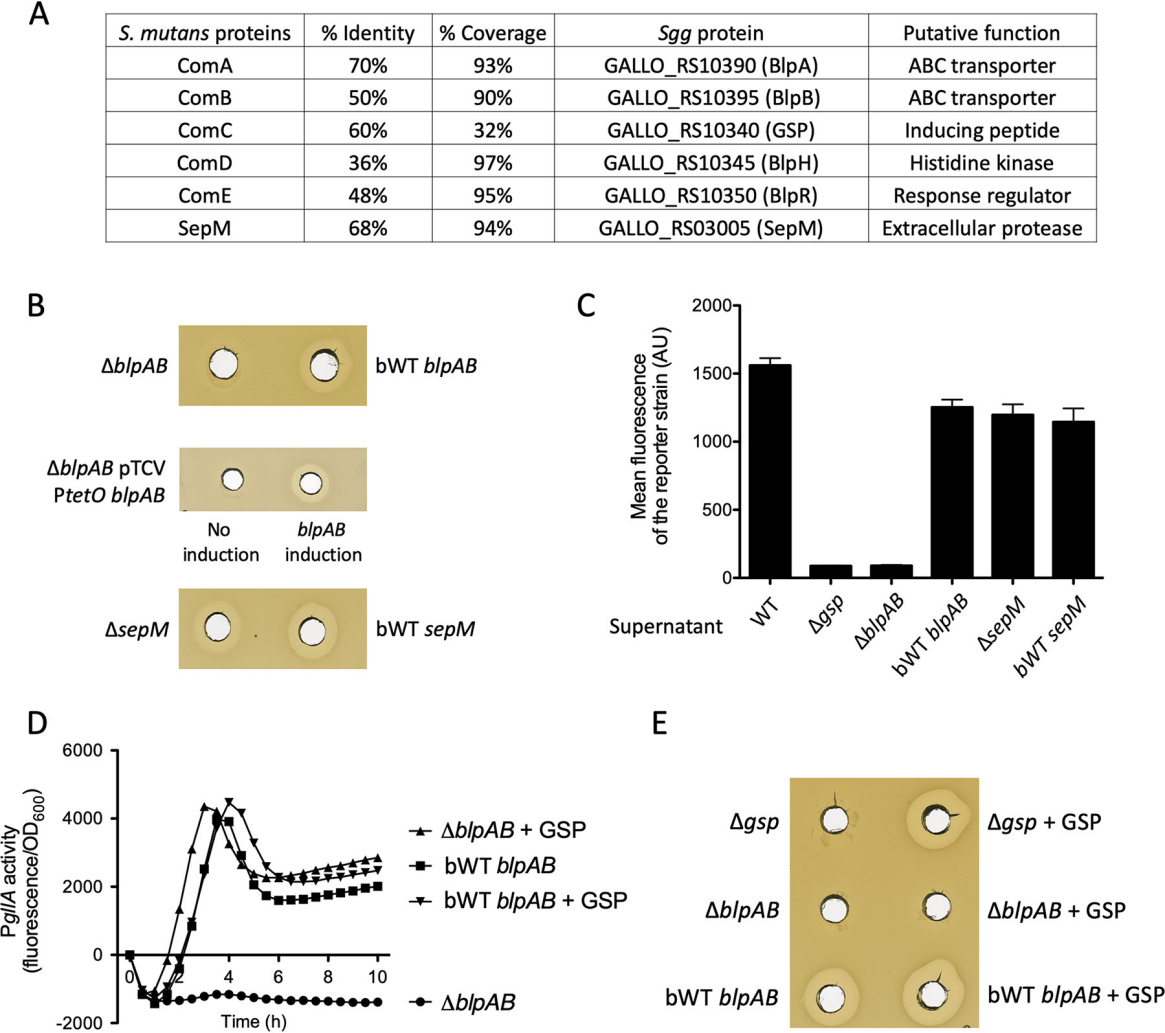
The ABC transporter BlpAB secretes both GSP and gallocin peptides. (A) Summary table of BLAST results comparing the ComABCDE system and SepM proteins of S. mutans with their homologous counterparts in S. gallolyticus subsp. *gallolyticus*. (B) Agar diffusion assay showing gallocin activity in the culture supernatant against S. gallolyticus subsp. *macedonicus*. Strains tested include S. gallolyticus subsp. *gallolyticus* UCN34 Δ*blpAB* and UCN34 Δ*sepM*, their bWT counterparts, and complemented S. gallolyticus subsp. *gallolyticus* UCN34 Δ*blpAB* containing the plasmid pTCVΩP*tetO*-*blpAB* with or without induction of P*tetO*. (C) Mean fluorescence of the reporter strain S. gallolyticus subsp. *gallolyticus* UCN34 Δ*gsp* pTCVΩP*gllA*-*gfp* resuspended in the supernatant of S. gallolyticus subsp. *gallolyticus* UCN34 WT, Δ*gsp*, Δ*blpAB*, bWT *blpAB*, Δ*sepM*, and bWT *sepM* strains. Results are means and standard deviations (SD) from three independent experiments. (D) P*gllA* activity in S. gallolyticus subsp. *gallolyticus* UCN34 Δ*blpAB* and its bWT counterpart containing the reporter plasmid pTCVΩP*gllA*-*gfp* with or without addition of 20 nM synthetic GSP. One representative curve of three independent experiments is shown here for each condition. (E) Agar diffusion assay showing gallocin activity in the culture supernatant against S. gallolyticus subsp. *macedonicus*. Strains tested include S. gallolyticus subsp. *gallolyticus* UCN34 Δ*gsp*, Δ*blpAB* and bWT *blpAB* cultivated with or without addition of 20 nM synthetic GSP.

We next aimed at determining how GSP was secreted and processed into a 21-mer peptide in S. gallolyticus subsp. *gallolyticus* UCN34. In S. mutans, the equivalent 21-mer MIP peptide encoded by *comC* is secreted by a specific ABC transporter composed of ComA and ComB proteins (Fig. 1A). Two genes (*gallo_rs10390* and *gallo_rs10395*) encoding a putative ABC transporter homologous to ComAB were found in the vicinity of the GSP and gallocin genes (Fig. 1A). To test the role of this putative transporter (named BlpAB) in GSP secretion and maturation, a mutant with an in-frame deletion of *gallo_rs10390/gallo_rs10395* was constructed in strain UCN34 and is referred to as UCN34 Δ*blpAB*. For each deletion mutant generated in S. gallolyticus subsp. *gallolyticus* clinical isolate UCN34, we also selected a clone that reverted back to the WT genotype (bWT) following homologous recombination. These bWT strains should display the WT phenotype and are isogenic to their mutant counterparts. Basic phenotypic characterization of several clones for a given mutation was carried out systematically (growth curve, immunofluorescence microscopy, and antibiotic resistance profile) to rule out any major secondary mutations that might have occurred during the engineering process. We then determined gallocin activity against a highly sensitive bacterium, Streptococcus gallolyticus subsp. *macedonicus*, using the supernatants of the Δ*blpAB* mutant compared to its bWT strain. As shown in Fig. 1B, gallocin activity was completely absent in the Δ*blpAB* mutant supernatant but was present in the bWT strain. Three possibilities can explain these results: (i) absence of GSP in the supernatant of the *ΔblpAB* mutant, (ii) absence of the two structural peptides (GllA1 and GllA2) constituting the active gallocin in the supernatant of the *ΔblpAB* mutant, or (iii) absence of both GSP and gallocin peptides in the supernatant of the *ΔblpAB* mutant.

To differentiate between these alternatives, we first tested the ability of the *ΔblpAB* supernatant to activate *gfp* transcription in the UCN34 Δ*gsp* pTCVΩP*gllA*-*gfp* reporter strain mentioned above. To do so, we resuspended the reporter strain in different supernatants. If GSP is present in the supernatant, the gallocin promoter will be activated, turning on GFP and allowing the bacteria to fluoresce. As shown in Fig. 1C, the reporter strain was not fluorescent in the absence of GSP (Todd-Hewitt broth with yeast extract [THY] only and Δ*gsp* strain supernatant) and became fluorescent in the presence of GSP (THY plus GSP and WT supernatant). No GFP fluorescence could be detected using the Δ*blpAB* strain supernatant, while a strong signal was detected in the bWT strain supernatant, demonstrating that GSP is not secreted in the Δ*blpAB* mutant, an observation that was verified by liquid chromatography-mass spectrometry (LC-MS) (Fig. 1C and Fig. S2). These results were further confirmed by introducing the reporter plasmid (pTCVΩP*gllA*-*gfp*) into the Δ*blpAB* mutant and measuring GFP fluorescence. The gallocin promoter was inactive in the Δ*blpAB* pTCVΩP*gllA*-*gfp* strain, while addition of synthetic GSP in the culture medium restored full gallocin promoter activity (Fig. 1D).

10.1128/mBio.03189-20.2 FIG S2 LC-MS of UCN34 Δ*blpAB* supernatant after 16 h incubation. No S. gallolyticus subsp. *gallolyticus* GSP was detected. Download FIG S2, PDF file, 0.4 MB. Copyright © 2021 Harrington et al. 2021 Harrington et al. This content is distributed under the terms of the Creative Commons Attribution 4.0 International license.

We next investigated whether the same ABC transporter could also secrete the gallocin GllA1 and GllA2 peptides, and therefore, we analyzed gallocin activity in the Δ*blpAB* mutant in the presence of synthetic GSP. As shown in Fig. 1D, addition of GSP to the culture medium restored gallocin production in the control Δ*gsp* mutant. However, no gallocin activity could be detected in the *ΔblpAB* mutant even in the presence of synthetic GSP, although the gallocin promoter was fully active under these conditions, as mentioned above.

### SepM is not involved in GSP maturation in S. gallolyticus subsp. *gallolyticus* UCN34.

In S. mutans, an extracellular protease, SepM, is responsible for CSP/MIP maturation by cleaving the three C-terminal amino acids of ComC after its secretion. BLAST analysis revealed that S. gallolyticus subsp. *gallolyticus* UCN34 possesses a close SepM homolog (Fig. 1A). Therefore, we reasoned that S. gallolyticus subsp. *gallolyticus* SepM could be involved in GSP maturation by cleaving the three N-terminal residues of the GSP 24-mer after secretion by the ABC transporter. To test this hypothesis, we deleted *sepM* in S. gallolyticus subsp. *gallolyticus* UCN34. As shown in Fig. 1B, gallocin production appears to be slightly reduced in the *ΔsepM* mutant compared to the isogenic bWT. To test whether the Δ*sepM* mutant was able to produce the fully active GSP 21-mer, we first analyzed the capacity of Δ*sepM* supernatants to induce gallocin promoter activity in the reporter strain Δ*gsp* pTCVΩP*gllA*-*gfp*. We found that the Δ*sepM* supernatant was able to fully activate the P*gllA* promoter in the reporter strain, suggesting that Δ*sepM* supernatants possess the active form of GSP. We then further assessed the Δ*sepM* strain supernatant using LC-MS and detected the presence of the mature 21-mer GSP (Fig. S3).

10.1128/mBio.03189-20.3 FIG S3 LC-MS of UCN34 Δ*sepM* supernatant after 16 h incubation. S. gallolyticus subsp. *gallolyticus* GSP expected: H_2_^+2^/2 (1246.6855 Da) and H_3_^+3^/3 (831.4594 Da). Download FIG S3, PDF file, 0.4 MB. Copyright © 2021 Harrington et al. 2021 Harrington et al. This content is distributed under the terms of the Creative Commons Attribution 4.0 International license.

**FIG 2:**
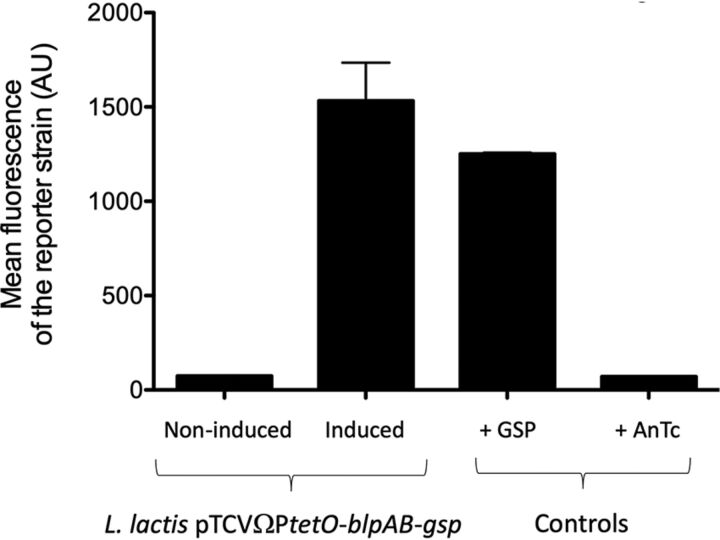
Mean fluorescence of the reporter strain S. gallolyticus subsp. *gallolyticus* UCN34 Δ*gsp* pTCVΩP*gllA*-*gfp* resuspended in the supernatant of Lactococcus lactis pTCVΩP*tetO-blpAB-gsp* produced with or without induction with anhydrotetracycline (200 ng/ml) or in THY supplemented with 100 nM synthetic GSP or 200 ng/ml anhydrotetracycline.

To further evaluate if a cell-bound protease is involved in GSP maturation, we incubated the GSP 24-mer, a form of GSP which has the residues KNK in addition to the residues in the 21-mer (referred to here as KNK-GSP), and the GSP 21-mer with washed UCN34 Δ*gsp* or Δ*sepM* cells in sterile saline solution for 30 min and analyzed the filtrates using LC-MS. Degradation of KNK-GSP to GSP was not observed following incubation with either UCN34 Δ*sepM* or UCN34 Δ*gsp* (Fig. S4 and S5). Both GSP and KNK-GSP were degraded to GSP-des-D1-L3 (an 18-mer GSP analog lacking the first three residues from the N terminus [D1, F2, and L3]), suggesting that another cell-bound protease exists in S. gallolyticus subsp. *gallolyticus* (Fig. S4 to S6). We wondered whether in S. gallolyticus subsp. *gallolyticus*, the specific BlpAB ABC transporter has a unique feature that allows it to process GSP directly to the mature GSP 21-mer, three residues past the double glycine leader sequence. To test this hypothesis, we introduced the *gsp* and *blpAB* genes in Lactococcus lactis under the control of an inducible promoter and evaluated the ability of the supernatants to activate the P*gllA* promoter in the reporter strain (S. gallolyticus subsp. *gallolyticus* Δ*gsp* pTCVΩP*gllA*-*gfp*). Indeed, the supernatant of the recombinant L. lactis strain induced with anhydrotetracycline was able to activate GFP production, while the control noninduced supernatant could not (Fig. 2). Furthermore, LC-MS analysis of the supernatants of L. lactis confirmed the presence of 21-mer GSP in the strain expressing the *gsp* and *blpAB* genes but not in the wild-type strain.

10.1128/mBio.03189-20.4 FIG S4 LC-MS of S. gallolyticus subsp. *gallolyticus* KNK-GSP incubated with UCN34 Δ*sepM* cells in saline solution for 30 min. KNK-GSP (P1) expected: P1H_4_^+4^/4 (716.4046 Da) and P1H_4_^+5^/5 (573.3251 Da). S. gallolyticus subsp. *gallolyticus* GSP-des-D1-L3 (P3) expected: P3H_3_^+3^/3 (706.3996 Da) and P1H_4_^+4^/4 (530.0515 Da). Download FIG S4, PDF file, 0.4 MB. Copyright © 2021 Harrington et al. 2021 Harrington et al. This content is distributed under the terms of the Creative Commons Attribution 4.0 International license.

10.1128/mBio.03189-20.5 FIG S5 LC-MS of S. gallolyticus subsp. *gallolyticus* KNK-GSP incubated with UCN34 Δ*gsp* cells in saline solution for 30 min. KNK-GSP expected: P1H_4_^+4^/4 (716.4046 Da) and P1H_5_^+5^/5 (573.3251 Da). GSP-des-D1-L3 expected: P3H_3_^+3^/2 (706.3996 Da) and P3H_4_^+^/4 (530.0515 Da). Download FIG S5, PDF file, 0.4 MB. Copyright © 2021 Harrington et al. 2021 Harrington et al. This content is distributed under the terms of the Creative Commons Attribution 4.0 International license.

Biswas and coworkers showed previously that the SepM proteases are quite promiscuous and can process CSP signals of other species (28). We wanted to confirm that the S. gallolyticus subsp. *gallolyticus* SepM is a functional protease; therefore, we repeated the washed-cell processing assays described above, but this time we added the S. mutans 21-mer CSP to washed UCN34 Δ*gsp* or UCN34 Δ*sepM* cells. The 18-mer CSP was found in filtrates treated with UCN34 Δ*gsp* but not in filtrates treated with UCN34 Δ*sepM*, indicating that S. gallolyticus subsp. *gallolyticus* SepM is functional and capable of processing inactive S. mutans 21-mer CSP into active 18-mer CSP (Fig. S7 and S8) (28). Last, we tested 21-mer S. gallolyticus subsp. *gallolyticus* GSP with S. mutans Δ*comC* washed cells. Surprisingly, we observed that S. mutans is capable of processing GSP to GSP-des-D1-L3 (Fig. S9).

10.1128/mBio.03189-20.6 FIG S6 LC-MS of S. gallolyticus subsp. *gallolyticus* GSP incubated with UCN34 Δ*gsp* cells in saline solution for 30 min. GSP (P2) expected: P2H_3_^+3^/3 (831.4594 Da), P2H_4_^+4^/4 (623.8464 Da) and P2H_5_^+^/5 (499.2786 Da). GSP-des-D1-L3 (P3) expected: P3H_3_^+3^/3 (706.3996 Da) and P3H_4_^+4^/4 (530.0515 Da). Download FIG S6, PDF file, 0.4 MB. Copyright © 2021 Harrington et al. 2021 Harrington et al. This content is distributed under the terms of the Creative Commons Attribution 4.0 International license.

10.1128/mBio.03189-20.7 FIG S7 LC-MS of S. mutans 21-mer CSP incubated with UCN34 Δ*gsp* cells in saline solution for 30 min. S. mutans 21-mer (P4) expected: P4H_2_^+2^/2 (1183.128 Da), P4H_3_^+3^/3 (789.0878 Da) and P4H_4_^+4^/4 (592.0676 Da). S. mutans 18-mer CSP (P5) expected: P5H_2_^+2^/2 (1034.0278 Da) and P4H_3_^+3^/3 (689.6876 Da). Download FIG S7, PDF file, 0.4 MB. Copyright © 2021 Harrington et al. 2021 Harrington et al. This content is distributed under the terms of the Creative Commons Attribution 4.0 International license.

10.1128/mBio.03189-20.8 FIG S8 LC-MS of S. mutans 21-mer CSP incubated with UCN34 Δ*sepM* cells in saline solution for 30 min. S. mutans 21-mer (P4) expected: P4H_2_^+2^/2 (1183.128 Da), P4H_3_^+3^/3 (789.0878 Da), and P4H_4_^+4^/4 (592.0676 Da). Download FIG S8, PDF file, 0.4 MB. Copyright © 2021 Harrington et al. 2021 Harrington et al. This content is distributed under the terms of the Creative Commons Attribution 4.0 International license.

10.1128/mBio.03189-20.9 FIG S9 LC-MS of S. gallolyticus subsp. *gallolyticus* GSP incubated with S. mutans Δ*comC* cells in saline solution for 30 min. GSP (P2) expected: P2H_3_^+3^/3 (831.4594 Da). GSP-des-D1-L3 (P3) expected: P3H_3_^+3^/3 (706.3996 Da) and P3H_4_^+4^/4 (530.0515 Da). Download FIG S9, PDF file, 0.4 MB. Copyright © 2021 Harrington et al. 2021 Harrington et al. This content is distributed under the terms of the Creative Commons Attribution 4.0 International license.

### Structure-activity relationships of S. gallolyticus subsp. *gallolyticus* GSP.

Our second aim in this study was to evaluate and determine the key structural motifs that drive GSP binding to its cognate histidine kinase, BlpH, thus activating gallocin gene transcription.

### (i) Alanine scan of S. gallolyticus subsp. *gallolyticus* GSP.

We first set out to perform a full alanine scan of the 21-mer GSP signal. Peptides were synthesized using standard solid-phase peptide synthesis (SPPS) conditions using a CEM Liberty1 microwave synthesizer on Cl-MPA Protide resin (LL). The peptides were then purified to homogeneity (>95%) using reverse-phase high-performance LC (RP-HPLC) and validated using mass spectrometry (for full experimental details and peptide characterization, see the supplemental material). The alanine scan revealed several residues that are important for receptor binding, as mutating them to alanine resulted in significant reduction in activity, yet no one residue was found to be critical for receptor activation, leading to the identification of a competitive inhibitor (Table 2). Specifically, the alanine scan revealed that the C-terminal half of the peptide (residues W10 to H20) was more important for receptor binding than the N-terminal half (residues D1 to D9), as modifications to the C-terminal half resulted in a significant decrease in potency, with the most important residue being W10 (Table 2).

**TABLE 2:** EC_50_s of S. gallolyticus subsp. *gallolyticus* GSP alanine analogs in UCN34 Δ*gsp*^*a*^

Compound	Sequence	EC_50_ (nM)^*b*^	95% CI^*c*^
GSP	DFLIVGPFDWLKKNHKPTKHA	2.96	1.70–5.14
GSP D1A	**A**FLIVGPFDWLKKNHKPTKHA	24.7	12.6–48.3
GSP F2A	D**A**LIVGPFDWLKKNHKPTKHA	19.9	8.79–45.3
GSP L3A	DF**A**IVGPFDWLKKNHKPTKHA	156	121–200
GSP I4A	DFL**A**VGPFDWLKKNHKPTKHA	137	61.5–307
GSP V5A	DFLI**A**GPFDWLKKNHKPTKHA	79.6	51.4–123
GSP G6A	DFLIV**A**PFDWLKKNHKPTKHA	276	138–553
GSP P7A	DFLIVG**A**FDWLKKNHKPTKHA	76.5	63.3–92.4
GSP F8A	DFLIVGP**A**DWLKKNHKPTKHA	397	248–635
GSP D9A	DFLIVGPF**A**WLKKNHKPTKHA	32.6	19.9–53.6
GSP W10A	DFLIVGPFD**A**LKKNHKPTKHA	>1,000	
GSP L11A	DFLIVGPFDW**A**KKNHKPTKHA	718	535–963
GSP K12A	DFLIVGPFDWL**A**KNHKPTKHA	299	259–346
GSP K13A	DFLIVGPFDWLK**A**NHKPTKHA	297	253–348
GSP N14A	DFLIVGPFDWLKK**A**HKPTKHA	93.5	61.4–142
GSP H15A	DFLIVGPFDWLKKN**A**KPTKHA	152	130–177
GSP K16A	DFLIVGPFDWLKKNH**A**PTKHA	893	515–1,550
GSP P17A	DFLIVGPFDWLKKNHK**A**TKHA	188	89.3–395
GSP T18	DFLIVGPFDWLKKNHKP**A**KHA	992	722–1,360
GSP K19A	DFLIVGPFDWLKKNHKPT**A**HA	106	64.4–175
GSP H20A	DFLIVGPFDWLKKNHKPTK**A**A	273	198–375

a See Materials and Methods for details of reporter strains and methods. All assays were performed in triplicate.

b EC_50_s were determined by testing peptides over a range of concentrations.

c CI, confidence interval.

### (ii) Truncation studies of S. gallolyticus subsp. *gallolyticus* GSP.

To further evaluate the roles of the N and C termini in receptor binding, and to determine the minimal sequence required for effective receptor activation, we conducted sequential truncations of the S. gallolyticus subsp. *gallolyticus* GSP signal from both ends. We also wanted to identify the residues in S. gallolyticus subsp. *gallolyticus* KNK-GSP that led to an ∼100-fold reduction in potency compared to GSP; therefore, we included in our analysis the sequential truncation of KNK-GSP to GSP. Starting with KNK-GSP, removal of a single residue (K) from the N terminus was sufficient to increase the potency of the peptide by 20-fold, resulting in an analog, NK-GSP, that was only ∼5-fold less potent than GSP (Table 3). Removal of an additional residue resulted in an analog, K-GSP, that was as potent as GSP.

**TABLE 3:** EC_50_s of elongated and truncated S. gallolyticus subsp. *gallolyticus* GSP analogs in UCN34 Δ*gsp*^*a*^

Compound	Sequence	EC_50_ (nM)^*b*^	95% CI^*c*^
KNK-GSP	**KNK**DFLIVGPFDWLKKNHKPTKHA	287	145–568
NK-GSP	**NK**DFLIVGPFDWLKKNHKPTKHA	14.1	8.01–24.7
K-GSP	**K**DFLIVGPFDWLKKNHKPTKHA	2.33	1.45–3.76
GSP-des-D1	FLIVGPFDWLKKNHKPTKHA	9.21	4.29–19.8
GSP-des-D1-F2	LIVGPFDWLKKNHKPTKHA	6.49	3.03–13.9
GSP-des-D1-L3	IVGPFDWLKKNHKPTKHA	2.22	1.41–3.52
GSP-des-D1-I4	VGPFDWLKKNHKPTKHA	3.80	2.49–5.80
GSP-des-D1-V5	GPFDWLKKNHKPTKHA	3.60	3.34–3.89
GSP-des-D1-G6	PFDWLKKNHKPTKHA	3.19	2.54–3.99
GSP-des-D1-P7	FDWLKKNHKPTKHA	7.21	3.79–13.7
GSP-des-D1-F8	DWLKKNHKPTKHA	39.7	21.4–73.6
GSP-des-D1-D9	WLKKNHKPTKHA	26.9	18.3–39.7
GSP-des-D1-W10	LKKNHKPTKHA	>1,000	
GSP-des-A21	DFLIVGPFDWLKKNHKPTKH	>1,000	
GSP-des-H20-A21	DFLIVGPFDWLKKNHKPTK	>1,000	
GSP-des-K19-A21	DFLIVGPFDWLKKNHKPT	>1,000	

a See Materials and Methods for details of reporter strains and methods. All assays were performed in triplicate.

b EC_50_s were determined by testing peptides over a range of concentrations.

c CI, confidence interval.

Moving to the native S. gallolyticus subsp. *gallolyticus* GSP, the importance of the C terminus was confirmed, as truncation of even a single residue led to loss of activity (Table 3). In contrast to what was observed with the C terminus, and to our surprise, we found that the N terminus is completely dispensable up to the seventh position (P7) and can accommodate the loss of up to nine residues without a significant reduction in potency (S. gallolyticus subsp. *gallolyticus* GSP-des-D1-D9 exhibited an EC_50_ of 26.9 nM, only 9-fold lower than S. gallolyticus subsp. *gallolyticus* GSP) (Table 3). Removal of the tenth residue (W10) resulted in a >35-fold reduction in potency compared to the analog lacking nine residues and a >330-fold reduction in potency compared to S. gallolyticus subsp. *gallolyticus* GSP.

## DISCUSSION

S. gallolyticus subsp. *gallolyticus* is an emerging human pathogen that utilizes quorum sensing (QS) to regulate the production of gallocin, a class IIb bacteriocin, to gain a competitive advantage over other commensals and colonize the colon under tumoral conditions (9). In this work and the accompanying paper (33), we set out to investigate and characterize the Blp-type QS circuitry in S. gallolyticus subsp. *gallolyticus*. In the accompanying paper, it was shown that a secreted peptide named GSP activates transcription of gallocin genes through a two-component system named BlpHR. In this work, we first set out to determine the maturation and export processes of the QS peptide, GSP. The *gsp* gene encodes a prepeptide of 45 amino acids harboring a classical N-terminal leader sequence ending with a double glycine. This leader sequence is generally cleaved right after the glycine doublet by an ABC transporter during secretion of the peptide. Thus, GSP was predicted to be a 24-mer pheromone. However, the secreted GSP was determined to be a 21-mer peptide, missing the first three N-terminal residues past the double glycine. Evaluating the potency of the two peptide variants revealed that the 21-mer GSP is about 100-fold more potent than the predicted 24-mer, suggesting that GSP maturation to a 21-mer peptide increases GSP efficiency compared to the 24-mer peptide. We first showed that in S. gallolyticus subsp. *gallolyticus*, GSP is not processed by the extracellular protease SepM, as is the CSP/MIP peptide in S. mutans (28, 29). In addition, the BlpAB transporter was shown to be sufficient to secrete the 21-mer GSP in L. lactis. Together, these results imply that the BlpAB transporter seems to have an atypical cleavage site for GSP, 3 residues after the predicted double glycine cleavage site. Therefore, the GSP 21-mer appears to be the product of BlpAB maturation rather than being processed by an extracellular protease. This observation is significant, as it challenges the double-glycine-leader dogma and highlights the need to confirm the structure of predicted signaling CSP and Blp peptides by isolating them from bacterial supernatants, rather than just predicting the cleavage site from their sequence using computational methods.

Further evaluation of the processing and maturation of the gallocin peptides revealed that the BlpAB transporter is also essential for gallocin peptide secretion. LC-MS analysis indicated that the gallocin prepeptides are cleaved right after the double glycine found in their leader sequences (data not shown).

Second, we performed the first comprehensive structure-activity relationship study of the GSP signal. Our alanine scan analysis revealed that replacement of either one of the side chain residues with alanine is detrimental to activity, with the C terminus being less tolerant of such modifications than the N terminus. However, no one residue was found to be critical to receptor binding (BlpH), since activity was reduced but not abolished in all the variants tested. This activity profile matches that of the CSP/MIP signal in S. mutans (34) and contrasts CSP signals of other streptococci, such as S. pneumoniae and Streptococcus mitis, where the N terminus is responsible for receptor activation, while hydrophobic residues in the central region are critical for initial receptor recognition (35–37). Furthermore, the sequential truncation analysis of the mature form of GSP (GSP-21) revealed that its first 9 N-terminal residues are dispensable for its activity, whereas the last 12 C-terminal residues are essential. This trend is again in contrast to other streptococcal CSPs, which generally exhibit a modifiable C terminus and a highly conserved N terminus (35, 37). The dispensability of the N terminus of the S. gallolyticus subsp. *gallolyticus* GSP is quite surprising, given the fact that it contains a negatively charged N-terminal residue (aspartate) similarly to the S. pneumoniae and S. mitis CSPs; however, in these two signaling molecules, the negatively charged N-terminal residue (glutamate) is responsible for receptor activation, and its modification leads to the formation of analogs capable of binding the receptor but not activating it (competitive inhibitors) (35–37).

Interestingly, it appears that removal of residues is more tolerated than replacement of the side chains of these residues with alanine, suggesting that although the N terminus does not play a significant role in receptor binding, modifications of this region may lead to conformational changes that result in steric clashes with the BlpH receptor and thus significantly lowered potency. Most striking, though, is the fact that nearly half of the GSP sequence (residues 1 to 9) can be removed without significantly affecting the peptide activity (9-fold reduction in potency compared to GSP). As the C-terminal region of GSP is more hydrophilic, whereas the N-terminal region is more hydrophobic, it is tempting to speculate that in the native environment the role of the N terminus is to increase the affinity of GSP to the bacterial cell membrane to prevent the peptide from diffusing away and allowing the C terminus to interact with the BlpH receptor. Overall, our findings revealed the minimal pheromone scaffold needed for gallocin activation and provide the groundwork for rationally designing peptide inhibitors targeting gallocin production.

## MATERIALS AND METHODS

### Chemicals.

All chemical reagents and solvents were purchased from Sigma-Aldrich or Chem-Impex and used without further purification. Double-distilled water (ddH_2_O) was purified using a Millipore analyzer feed system. Solid-phase Cl-MPA Protide resin was purchased from CEM Corporation. 9-Fluorenylmethoxycarbonyl (Fmoc)-protected l-α-amino acids were purchased from Advanced ChemTech.

### Instrumentation.

Reverse-phase high-performance liquid chromatography (RP-HPLC) was performed using a Shimadzu UFLC system equipped with a CBM-20A communications bus module, two LC-20AT pumps, a SIL-20A auto sampler, a SPD-20A UV–visible-light (UV/Vis) detector, a CTO-20A column oven, and a FRC-10A fraction collector. All RP-HPLC solvents (ddH_2_O and HPLC-grade acetonitrile [ACN]) contained 0.1% trifluoroacetic acid (TFA). Preparative RP-HPLC was performed using a Phenomenex Kinetex 5-μm, 100-Å C_18_ column (250 by 10 mm), while analytical RP-HPLC was performed using a Phenomenex Kinetex 5-μm, 100-Å C_18_ column (250 by 4.6 mm). Fmoc-based solid-phase peptide synthesis was performed on a Discover Microwave and Liberty1 automated peptide synthesizer (CEM Corp.). Matrix-assisted laser desorption ionization–time-of-flight mass spectrometry (MALDI-TOF MS) data were obtained by mixing 0.75 μl of sample with 0.75 μl of matrix solution (α-cyano-4-hydroxycinnamic acid dissolved in ddH_2_O-ACN [1:1] with 0.1% TFA) on an MSP 96 polished steel target plate (Bruker Daltonics) and allowing it to air dry. Data were obtained using a Bruker Microflex spectrometer equipped with a 60 Hz (337-nm wavelength) nitrogen laser and a reflectron. MALDI-TOF MS data were obtained using reflectron positive ion mode with the following settings: ion source 1, 19 kV; ion source 2, 15.9 kV; lens, 8.75 kV; reflector, 20 kV; up to 300 Da matrix suppression; 200 laser shots per sample; and detector gain, 1,594 V. Exact mass data were obtained on an Agilent Technologies 6230 time-of-flight mass spectrometer (TOF-MS) with the following settings for positive electrospray ionization mode (ESI+): capillary voltage, 3,500 V; fragmentor voltage, 175 V; skimmer voltage, 65 V; octopole radio frequency voltage, peak power point (Oct 1 RF Vpp), 750 V; gas temperature, 325°C; drying gas flow rate, 3 liters/min; and nebulizer, 25 lb/in^2^.

### Bacterial growth conditions.

The S. gallolyticus subsp. *gallolyticus* strains used in this study were UCN34 (wild type), UCN34 Δ*gallo_rs10340* (Δ*gsp*), UCN34 Δ*gsp* pTCVΩP*gllA*-*gfp*, UCN34 Δ*gallo_rs03005* (Δ*sepM*), UCN34 Δ*sepM* pTCVΩP*gllA*-*gfp*, UCN34 Δ*gallo_rs10390/10395* (Δ*blpAB*), UCN34 Δ*blpAB* pTCVΩP*gllA*-*gfp*; L. lactis NZ9000 pTCVΩP*tetO-blpAB-gsp* was also used. The following procedure was followed for each S. gallolyticus subsp. *gallolyticus* isolate: a freezer stock was streaked onto a plate of Todd-Hewitt agar supplemented with 0.5% yeast extract (THY plate). Strains containing the pTCVΩP*gllA*-*gfp* plasmid were grown in the presence of erythromycin (EM) at a final concentration of 10 μg/μl. The plates were incubated for 12 to 24 h in a CO_2_ incubator (37°C with 5% CO_2_). Fresh colonies were picked, inoculated into a sterilized culture tube containing 2 ml of sterile THY broth, and incubated statically in a CO_2_ incubator overnight. The overnight culture was used for the experiments described below. L. lactis strains were grown under similar conditions, with the exception of the absence of CO_2_ and a growth temperature of 30°C.

### Solid-phase peptide synthesis.

Peptide synthesis and purification of analogs was conducted using previously established methods (10). All peptides were purified to homogeneity (>95%), and their identity was validated via mass spectrometry (Table S1).

10.1128/mBio.03189-20.10 TABLE S1 MS and HPLC data for S. gallolyticus subsp. *gallolyticus* GSP analogs. Download Table S1, PDF file, 0.1 MB. Copyright © 2021 Harrington et al. 2021 Harrington et al. This content is distributed under the terms of the Creative Commons Attribution 4.0 International license.

### General assay considerations.

Fluorescence and absorbance measurements were recorded using a Biotek Synergy H1 microplate reader using Gen5 data analysis software (v. 3.03). Biological assays were performed in triplicate per trial. EC_50_s from three trials were calculated using GraphPad Prism software (v. 7.0) using a sigmoidal curve fit.

Stock solutions of peptides (1 mM) were prepared in dimethyl sulfoxide (DMSO) and stored at 4°C in sealed glass vials. The maximum concentration of DMSO used in all biological assays did not exceed 2% (vol/vol). Black polystyrene 96-well microtiter plates (Costar) were used for the GFP cell-based reporter assays.

### Gallocin induction reporter gene assay protocol.

Peptide stock solutions were serially diluted with DMSO in either 1:2, 1:3, or 1:5 dilutions, and 2 μl of the diluted solution was added to each of the wells in a black 96-well microtiter plate. Each concentration was tested in triplicate, with DMSO only used as a negative control. Screening of peptide analogs was conducted using the S. gallolyticus subsp. *gallolyticus* UCN34 Δ*gsp* pTCVΩP*gllA*-*gfp* strain. An overnight culture was used to make a fresh 2-ml culture without EM using a 1:10 dilution on the day of the experiment and incubated in a CO_2_ incubator (37°C with 5% CO_2_) for 3 to 4 h to reach mid-log to late log phase of growth (OD_600_ = 0.6 to 0.9). A final 1:10 dilution was made on a larger scale (20 ml) without EM and used for the assay, where 198 μl diluted bacterial culture was added to each well of the microtiter plate containing peptides. Plates were incubated at 37°C with shaking at 200 rpm. Fluorescence (excitation, 485 nm; emission, 516 nm) and optical density at 600 nm (OD_600_) readings were recorded for each well using a plate reader. Measurements were taken 1 h after inoculation and were recorded every 20 to 30 min for up to 3 h to capture the maximum fluorescence signal. The maximum fluorescence signal was normalized with the OD_600_ value and used to construct dose curves to determine the EC_50_.

### Analysis of SepM function by LC-MS experiments.

S. gallolyticus subsp. *gallolyticus* strains (Δ*gsp* and Δ*sepM*) and S. mutans SMCC3 (Δ*comC*) were grown in 60 ml THY overnight at 37°C with 5% CO_2_ and centrifuged at 4,000 × *g* for 15 min. The supernatant was discarded, and the cell pellets were washed with 30 ml of sterile saline solution (0.9% [wt/vol] NaCl). The cell suspensions were centrifuged at 4,000 × *g* for 15 min, and the supernatant was discarded. The cell pellet was resuspended in 6 ml sterile saline solution and used for the SepM function experiment. The peptides tested (S. gallolyticus subsp. *gallolyticus* GSP, S. gallolyticus subsp. *gallolyticus* KNK-GSP, and S. mutans 21-mer CSP) were resuspended in sterile H_2_O to a final concentration of 1 mM. For each strain tested, around 900 μl of the cell suspension was mixed with 100 μl of each peptide separately to give a 100 μM concentration. The cell-only control for each strain was made with 900 μl of each cell suspension with 100 μl of sterile saline solution. The peptide-only control for each peptide was made in sterile saline solution at a 100 μM concentration. All variables were tested in sterile 1.5-ml microcentrifuge tubes. All samples were incubated at 37°C with shaking at 200 rpm for 30 min. After 30 min, the samples were centrifuged at 5,000 × *g* for 1 min. A 200-μl aliquot from each sample was filtered through a sterile 0.45-μm syringe filter (Phenomenex Phenex-RC 4-mm syringe filter) into a sterile 1.5-ml microcentrifuge tube. LC-MS analysis of each sample was performed using XBridge C_18_ columns (5 μm, 4.6 by 150 mm) on an Agilent Technology 1200 series LC connected to Agilent Technologies 6230 TOF-MS. The solvents used for LC-MS were as follows: mobile phase A, ddH_2_O plus 0.1% formic acid, and mobile phase B, ACN plus 0.1% formic acid. The LC-MS method used to analyze components in the filtrates had an injection volume of 100 μl and a linear gradient of 5% to 95% B over 25 min, with a flow rate of 0.5 ml/min.

### LC-MS experiments identifying secreted GSP pheromone.

S. gallolyticus subsp. *gallolyticus* strains (wild type, Δ*sepM* mutant, and Δ*blpAB* mutant) were grown in 40 ml of sterile THY at 37°C with 5% CO_2_ for 12 h. The cultures were centrifuged at 4,000 × *g* for 15 min, the supernatant was discarded, and the cell pellets were resuspended in 5 ml of sterile THY. The resuspended cells were incubated for 16 h and centrifuged at 4,000 × *g* for 15 min. The supernatant was filter sterilized using a 0.22-μm syringe filter (CELLTREAT PES 30-mm diameter syringe filter) in a sterile 1.5-ml microcentrifuge tube. LC-MS analysis of each sample was performed using an XBridge C_18_ column (5 μm; 4.6 by 150 mm) on an Agilent Technology 1200 series LC connected to an Agilent Technologies 6230 TOF-MS. The solvents used for LC-MS were as follows: mobile phase A, ddH_2_O plus 0.1% formic acid, and mobile phase B, ACN plus 0.1% formic acid. The LC-MS method used to analyze components in the filtrates had an injection volume of 50 μl and a linear gradient of 5% to 95% B over 25 min, with a flow rate of 0.5 ml/min. L. lactis pTCVΩP*tetO-blpAB-gsp* was inoculated from a fresh plate into 40 ml of sterile THY containing 200 ng/ml anhydrotetracycline to an initial OD_600_ of 0.1, and grown at 30°C for 1 h with shaking at 200 rpm. Cultures were then centrifuged at 5,000 × *g* for 15 min, after which the supernatant was filter sterilized using a 0.22-μm syringe filter (CELLTREAT PES 30-mm diameter syringe filter) in a sterile 50-ml centrifuge tube. Ammonium sulfate (60% [wt/vol]) was added to the filtered supernatants to facilitate precipitation of the peptide. Supernatants were incubated with ammonium sulfate for 1 h at 4°C and centrifuged at 5,000 × *g* for 15 min. The supernatant was discarded, and pelleted proteins were resuspended in 10 ml 50:50 acetonitrile-water, frozen, and lyophilized for 24 h. Following lyophilization, LC-MS analysis was performed on precipitated components of the L. lactis supernatant, using an injection volume of 50 μl and a linear gradient of 5% to 95% B over 50 min, with a flow rate of 0.25 ml/min.

### Bacteriocin activity assay.

For well diffusion assays, 2 ml of prey bacteria (here, exponentially growing S. gallolyticus subsp. *macedonicus*; OD_600_ ≈ 0.5) were poured on a THY agar plate. After removal of excess liquid, the plate was dried for 15 min. Using sterile tips, 5-mm-diameter wells were dug into the agar. Wells were filled with 80 μl of S. gallolyticus subsp. *gallolyticus* filtered supernatant supplemented with 0.1% Tween 20. When the wells were dry, plates were incubated inverted overnight at 37°C, and the inhibition rings were observable the next day.

### Construction of deletion mutants in S. gallolyticus subsp. *gallolyticus* strain UCN34.

The Δ*blpAB* (*gallo_rs10390-gallo_rs10395*) and Δ*sepM* (*gallo_rs03005*) mutants were constructed as reported previously in S. gallolyticus subsp. *gallolyticus* strain UCN34 (38). The primers used are listed in Table 4. Briefly, a 1-kb fragment corresponding to the 5′ and 3′ ends of the region to delete was obtained by splicing by overlap extension PCR, digested with the restriction enzymes EcoRI and BamHI, and cloned into the thermosensitive vector pG1-oriT_TnGBS1_. The resulting plasmids, pG1Ω*blpAB* and pG1Ω*sepM*, were electroporated in Streptococcus agalactiae NEM316 and transferred to S. gallolyticus subsp. *gallolyticus* UCN34 by conjugation. Chromosomal integration of the plasmid was selected on THY containing erythromycin (10 μg/ml) at 38°C, a nonpermissive temperature for plasmid replication. Then, excision of the plasmid from the chromosome by a second event of homologous recombination was obtained by successive cultures at 30°C in THY broth without erythromycin, resulting in either gene deletion or reversion back to the wild type (bWT). Mutants were systematically tested for erythromycin sensitivity to confirm the loss of the plasmid and confirmed by PCR and sequencing of the chromosomal locus flanking the deletion region.

**TABLE 4:** Primers used in this study

Use and primer	Sequence (5′ to 3′)^*a*^	Fragment length (bp)
*blpAB* deletion		
Del *blpAB*-1	TTCT**GAATTC**GTCTAACAGATTGTGAGG	571
Del *blpAB*-2	ATGGCTGGACTTATCATCTTCTCATAACCTTTCCC	
Del *blpAB*-3	GGGAAAGGTTATGAGAAGATGATAAGTCCAGCCAT	552
Del *blpAB*-4	TTCT**GGATCC**AACGCCTGCGGTGAGTGA	
*sepM* deletion		
Del *sepM*-1	TTCT**GAATTC**CGGGGTCTTTTGACCCTG	513
Del *sepM*-2	TCGTAGATAGTCAATTGCGCGATATAAAATACGACC	
Del *sepM*-3	GGTCGTATTTTATATCGCGCAATTGACTATCTACGA	507
Del *sepM*-4	TTCT**GGATCC**AATTAGCACATTTGCACC	
pTCVΩP*tetO*-*blpAB_gsp*		
*blpAB-Bam*H1	TTCT**GGATCC**AAAAGGGAAAGGTTATGA	3,568
*blpAB-Pst*I	TTCT**CTGCAG**TCAAAAATCTTTTAATAG	
*gsp-Pst*I	TTCT**CTGCAG**CACTAAGGAGGTTTATAAA	161
*gsp*-*Sph*I	TTCT**GCATGC**GGTTTAAGCGTGTTTAGT	

a Enzyme restriction sites are in bold.

### Construction of L. lactis NZ9000 pTCVΩP*tetO-blpAB-gsp*.

Plasmid construction was performed as described in the accompanying paper (33). Briefly, *blpAB* and *gsp* genes were amplified by PCR using the primers listed in Table 4. They were cloned by digestion-ligation in the pTCV-P*tetO* vector to obtain pTCVΩP*tetO*-*blpAB-gsp*. This vector was transformed and amplified in competent *E.coli* TOP10 cells (Thermo Fisher), verified by sequencing and finally transformed by electroporation in L. lactis NZ9000.

### Indirect detection of GSP in the supernatant.

Strains to be tested for GSP production (S. gallolyticus subsp. *gallolyticus* strains or L. lactis pTCVΩP*tetO-blpAB-gsp*) were inoculated at an OD_600_ of 0.1 in THY and grown at 37°C (30°C for L. lactis). At an OD_600_ of 1, the cells were pelleted and the supernatants were harvested and filtered in order to test for the presence of GSP. After overnight incubation, 100 μl of the reporter strain S. gallolyticus subsp. *gallolyticus* Δ*gsp* pTCVΩP*gllA*-*gfp* was pelleted and resuspended in 1 ml of the tested supernatant. After 3 h of incubation at 37°C, the fluorescence of the reporter strain was measured by flow cytometry using a MACSQuant VYB flow cytometer.
