# Supplementary material for: Secretion, Maturation, and Activity of a Quorum Sensing Peptide (GSP) Inducing Bacteriocin Transcription in Streptococcus gallolyticus
Source: mBio. 2021 Jan 5;12(1):e03189-20. doi: 10.1128/mBio.03189-20 (PMC8545107; doi:10.1128/mBio.03189-20)
Supplement: FIG S1 [file mbio.03189-20-sf001.pdf]

LC-MS identifying secreted GSP pheromone

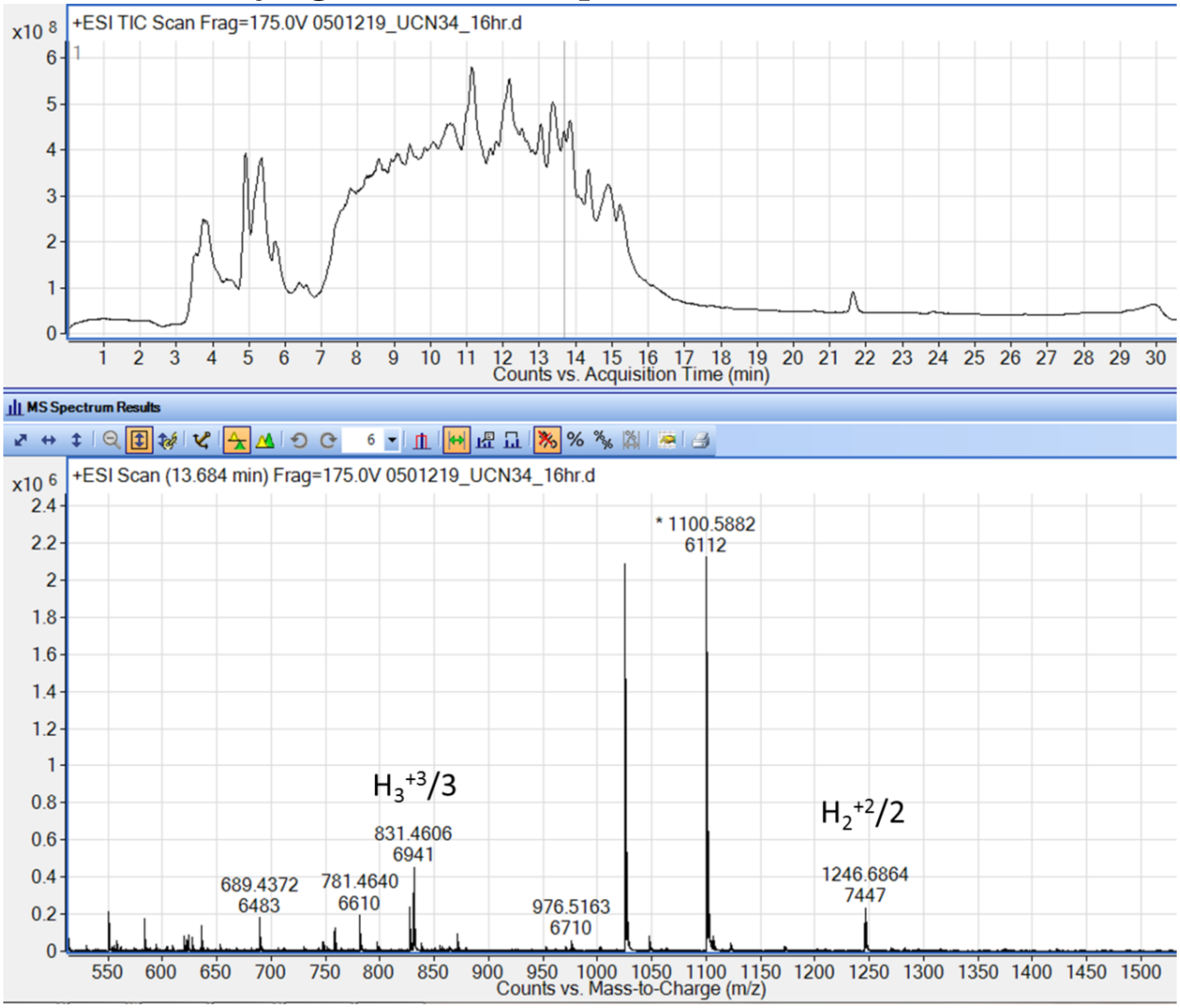

Figure S1. LC-MS of UCN34 supernatant after 16 h incubation. *Sgg* GSP expected: $H_2^{+2}/2$ [1246.6855 Da] and $H_3^{+3}/3$ [831.4594 Da].
