# Supplementary figures and images for: Secretion, Maturation, and Activity of a Quorum Sensing Peptide (GSP) Inducing Bacteriocin Transcription in Streptococcus gallolyticus
Source: mBio. 2021 Jan 5;12(1):e03189-20. doi: 10.1128/mBio.03189-20 (PMC8545107; doi:10.1128/mBio.03189-20)

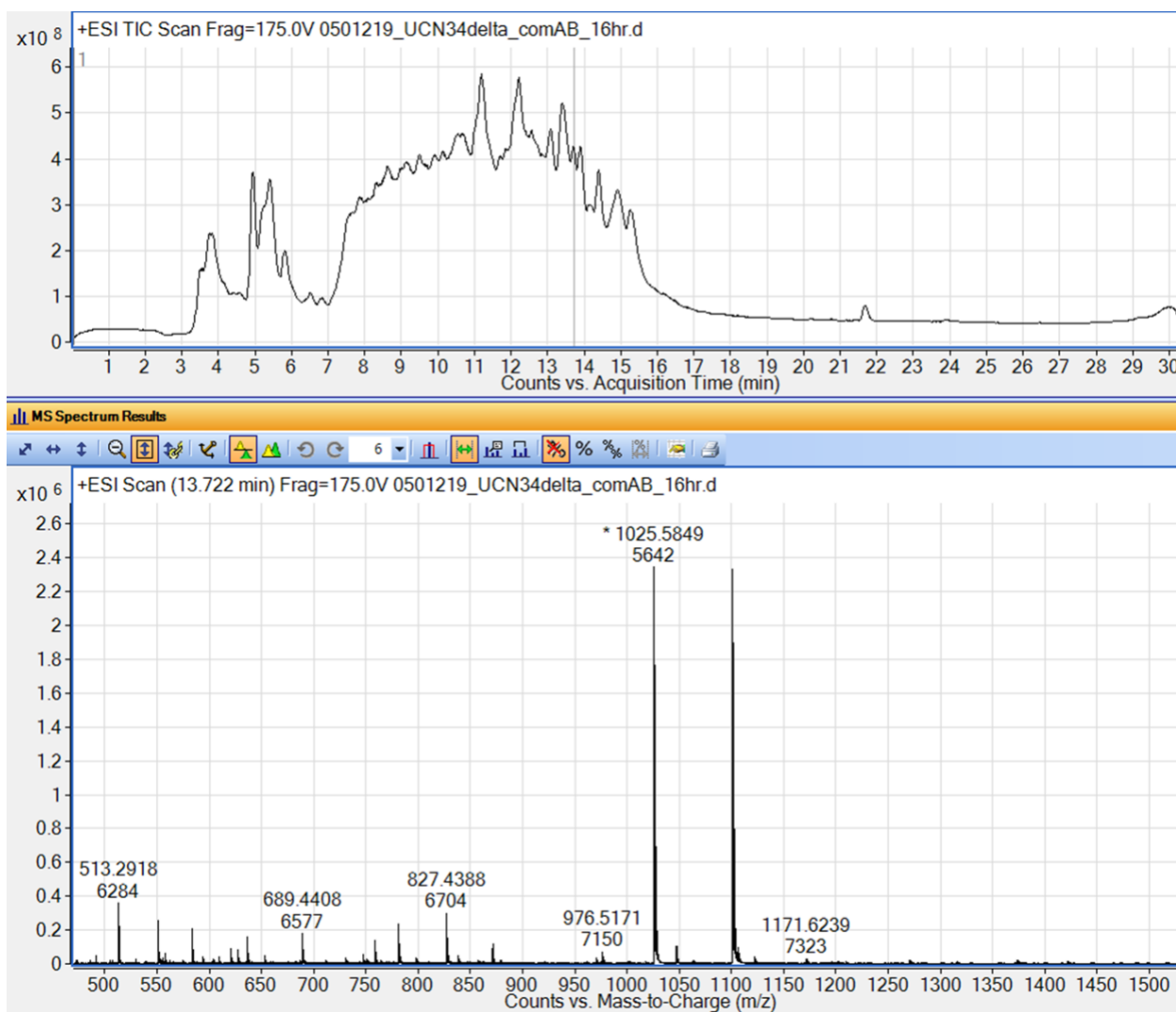

**Figure S2.** LC-MS of UCN34 $\Delta$ *blpAB* supernatant after 16 h incubation. No *Sgg* GSP is detected.

Supplement: FIG S2 [file mbio.03189-20-sf002.pdf]
