# Supplementary material for: Secretion, Maturation, and Activity of a Quorum Sensing Peptide (GSP) Inducing Bacteriocin Transcription in Streptococcus gallolyticus
Source: mBio. 2021 Jan 5;12(1):e03189-20. doi: 10.1128/mBio.03189-20 (PMC8545107; doi:10.1128/mBio.03189-20)
Supplement: FIG S4 [file mbio.03189-20-sf004.pdf]

LC-MS results of SepM Studies

KNK-*Sgg* GSP = P1

Sgg GSP-des-D1-L3 = P3

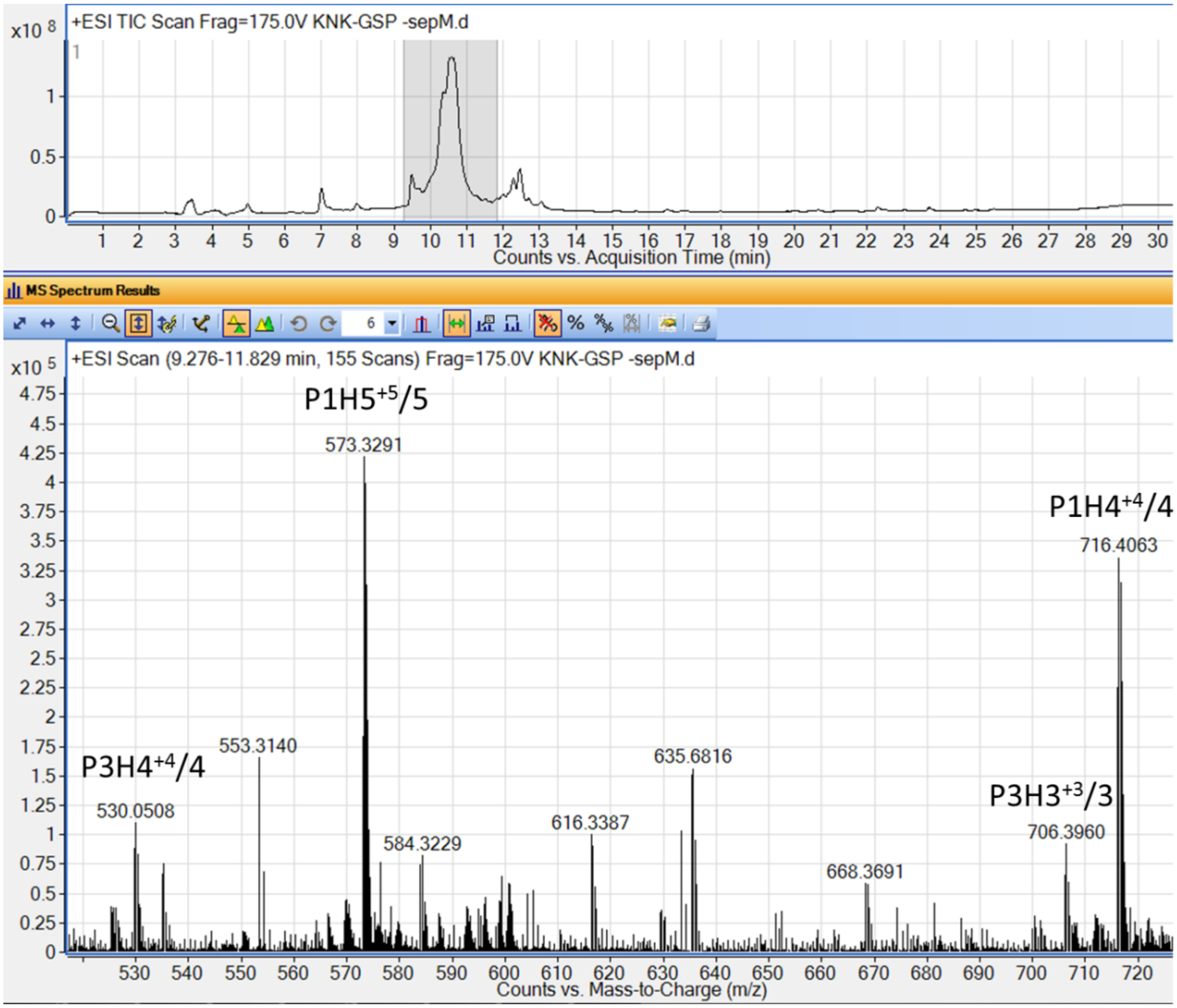

Figure S4. LC-MS of KNK-*Sgg* GSP incubated with UCN34Δ*sepM* cells in saline solution for 30 min. KNK-*Sgg* GSP (P1) expected: $P1H_4^{+4}/4$ [716.4046 Da] and $P1H_4^{+5}/5$ [573.3251 Da]. *Sgg* GSP-des-D1-L3 (P3) expected: $P3H_3^{+3}/3$ [706.3996 Da] and $P1H_4^{+4}/4$ [530.0515 Da].
