# Supplementary material for: Secretion, Maturation, and Activity of a Quorum Sensing Peptide (GSP) Inducing Bacteriocin Transcription in Streptococcus gallolyticus
Source: mBio. 2021 Jan 5;12(1):e03189-20. doi: 10.1128/mBio.03189-20 (PMC8545107; doi:10.1128/mBio.03189-20)
Supplement: FIG S8 [file mbio.03189-20-sf008.pdf]

S. mutans 21-mer CSP = P4

S. mutans 18-mer CSP = P5

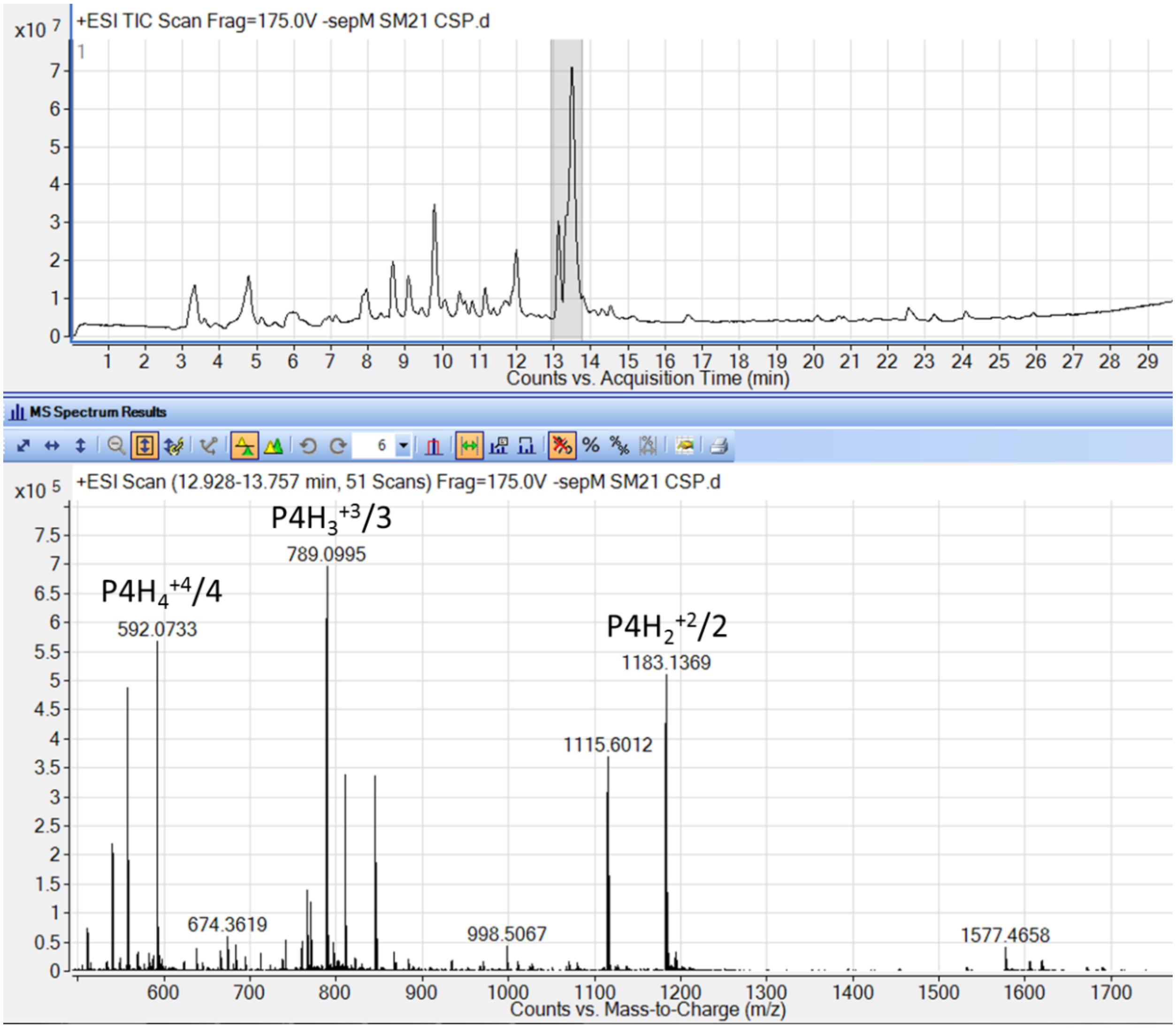

Figure S8. LC-MS of *S. mutans* 21-mer CSP incubated with UCN34Δ*sepM* cells in saline solution for 30 min. *S. mutans* 21-mer (P4) expected: $P4H_2^{+2}/2$ [1183.128 Da], $P4H_3^{+3}/3$ [789.0878 Da] and $P4H_4^{+4}/4$ [592.0676 Da].
