# Supplementary material for: Secretion, Maturation, and Activity of a Quorum Sensing Peptide (GSP) Inducing Bacteriocin Transcription in Streptococcus gallolyticus
Source: mBio. 2021 Jan 5;12(1):e03189-20. doi: 10.1128/mBio.03189-20 (PMC8545107; doi:10.1128/mBio.03189-20)
Supplement: FIG S9 [file mbio.03189-20-sf009.pdf]

Sgg GSP = P2

Sgg GSP-des-D1-L3 = P3

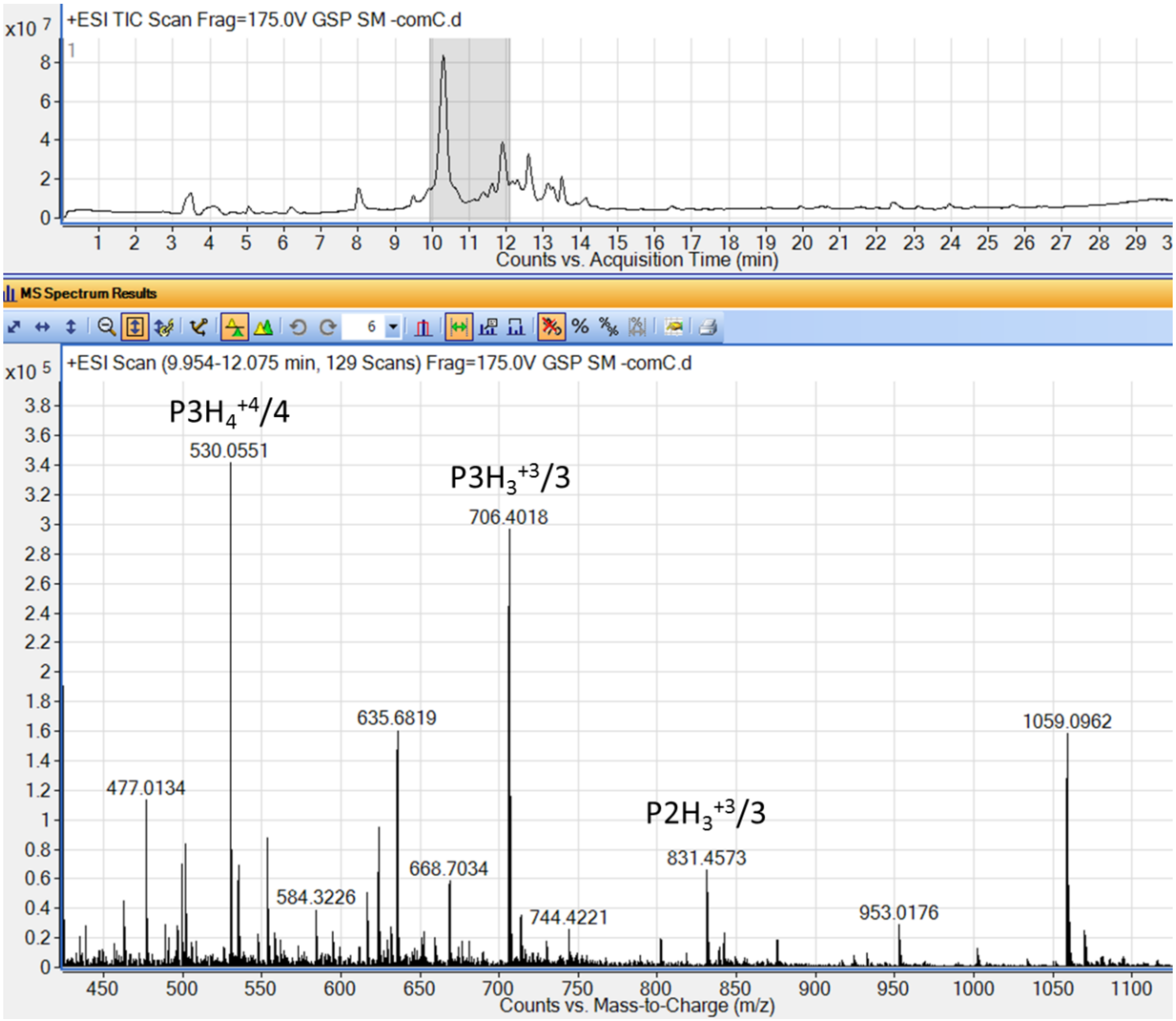

Figure S9. LC-MS of *Sgg* GSP incubated with *S. mutans* Δ*comC* cells in saline solution for 30 min. *Sgg* GSP (P2) expected: $P2H_3^{+3}/3$ [831.4594 Da]. *Sgg* GSP-des-D1-L3 (P3) expected: $P3H_3^{+3}/3$ [706.3996 Da] and $P3H_4^{+4}/4$ [530.0515 Da].
