# Supplementary material for: Secretion, Maturation, and Activity of a Quorum Sensing Peptide (GSP) Inducing Bacteriocin Transcription in Streptococcus gallolyticus
Source: mBio. 2021 Jan 5;12(1):e03189-20. doi: 10.1128/mBio.03189-20 (PMC8545107; doi:10.1128/mBio.03189-20)
Supplement: TABLE S1 [file mbio.03189-20-st001.pdf]

<u>MS and HPLC data of *Sgg* GSP analogs</u>

Table S1. MS and HPLC data of *Sgg* GSP analogs

Compound Name	Calc. EM $[MH_3]^{+3}/3$	Obs. EM $[MH_3]^{+3}/3$	Purity (%)
Sgg GSP D1A	816.7962	816.7961	> 99
Sgg GSP F2A	806.1157	806.1149	> 96
Sgg GSP L3A	817.4438	817.4423	> 98
Sgg GSP I4A	817.4438	817.4420	> 99
Sgg GSP V5A	822.1157	822.1125	> 99
Sgg GSP G6A	836.1313	836.1272	> 99
Sgg GSP P7A	617.3425 $[MH_4]^{+4}/4$	617.3395 $[MH_4]^{+4}/4$	> 99
Sgg GSP F8A	806.1157	806.1132	> 99
Sgg GSP D9A	816.7962	816.7952	> 99
Sgg GSP W10A	793.1120	793.1087	> 97
Sgg GSP L11A	817.4438	817.4406	> 99
Sgg GSP K12A	812.4402	812.4377	> 98
Sgg GSP K13A	812.4402	812.4396	> 99
Sgg GSP N14A	817.1242	817.1244	> 97
Sgg GSP H15A	809.4522	809.4504	> 99
Sgg GSP K16A	812.4402	812.4375	> 99
Sgg GSP P17A	822.7876	822.7867	> 98
Sgg GSP T18A	616.3438 $[MH_4]^{+4}/4$	616.3413 $[MH_4]^{+4}/4$	> 95
Sgg GSP K19A	609.5819	609.5796	> 99
Sgg GSP H20A	809.4522	809.4485	> 98
KNK-*Sgg* GSP	716.4046 $[MH_4]^{+4}/4$	716.4021 $[MH_4]^{+4}/4$	> 99
NK-*Sgg* GSP	912.1721	912.1726	> 98
K-*Sgg* GSP	874.1578	874.1547	> 97
Sgg GSP-des-D1	595.0897 $[MH_4]^{+4}/4$	595.0869 $[MH_4]^{+4}/4$	> 97
Sgg GSP-des-D1F2	558.3226 $[MH_4]^{+4}/4$	558.3211 $[MH_4]^{+4}/4$	> 96
Sgg GSP-des-D1-L3	706.3996	706.3964	> 99
Sgg GSP-des-D1-I4	668.7049	668.7026	> 98
Sgg GSP-des-D1-V5	635.6821	635.6801	> 96
Sgg GSP-des-D1-G6	616.6750	616.6745	> 99
Sgg GSP-des-D1-P7	583.9898	583.9899	> 97
Sgg GSP-des-D1-F8	801.9468 $[MH_2]^{+2}/2$	801.9448 $[MH_2]^{+2}/2$	> 98
Sgg GSP-des-D1-D9	744.4333 $[MH_2]^{+2}/2$	744.4365 $[MH_2]^{+2}/2$	> 96
Sgg GSP-des-D1-W10	434.5982	434.5997	> 98
Sgg GSP-des-A21	807.7804	807.7769	> 96
Sgg GSP-des-H20A21	762.0941	762.0955	> 98
Sgg GSP-des-K19-A21	719.3958	719.3952	> 99

EM = Exact Mass
